# The triptolide derivative MRx102 inhibits Wnt pathway activation and has potent anti-tumor effects in lung cancer

**DOI:** 10.1186/s12885-016-2487-7

**Published:** 2016-07-11

**Authors:** Theresa A. Reno, Sun-Wing Tong, Jun Wu, John M. Fidler, Rebecca Nelson, Jae Y. Kim, Dan J. Raz

**Affiliations:** Division of Thoracic Surgery, City of Hope Medical Center, 1500 E. Duarte Rd., Duarte, CA 91010 USA; Department of Pathology, City of Hope Medical Center, Duarte, CA USA; Division of Comparative Medicine, Beckman Research Institute, City of Hope Medical Center, Duarte, CA USA; MyeloRx LLC, Vallejo, CA USA; Department of Biostatistics, City of Hope Medical Center, Duarte, CA USA

**Keywords:** MRx102, Triptolide, Lung cancer, Metastasis, Wnt

## Abstract

**Background:**

The natural compound triptolide has been shown to decrease cell proliferation and induce apoptosis and cellular senescence. We previously demonstrated that triptolide decreases tumor formation and metastasis of human non-small cell lung cancer cells (NSCLC). Due to the toxicity of triptolide, derivatives of the natural compound have been developed that show more favorable toxicity profiles and pharmacokinetics in animal models. The purpose of this study was to evaluate MRx102 as a novel therapeutic for lung cancer.

**Methods:**

Mice injected subcutaneously with H460 lung cancer cells were treated with MRx102 or carboplatin to determine the effect of MRx102 on tumor formation in comparison to standard treatment. Patient-derived xenografts (PDX) with different WIF1 expression levels were treated with MRx102 or cisplatin. We tested the effects of MRx102 treatment on migration and invasion of lung cancer cells using Transwell filters coated with fibronectin and Matrigel, respectively. Tail vein injections using H460 and A549 cells were performed.

**Results:**

Here we report that the triptolide derivative MRx102 significantly decreases NSCLC proliferation and stimulates apoptosis. Further, MRx102 potently inhibits NSCLC haptotactic migration and invasion through Matrigel. In vivo, NSCLC tumor formation and metastasis were greatly decreased by MRx102 treatment. The decrease in tumor formation by MRx102 in the patient-derived xenograft model was WIF1-dependent, demonstrating that MRx102 is a potent inhibitor of the Wnt pathway in low WIF1 expressing NSCLC patient tumors.

**Conclusions:**

These results indicate that MRx102 has potent antitumor effects both in vitro and in vivo, and is a potential novel therapy for the treatment of NSCLC.

## Background

Lung cancer is the leading cause of cancer-related deaths world-wide in both men and women [1]. The carcinogenic toxins in cigarette smoke that create inflammation and accumulation of somatic mutations in the cellular DNA have been implicated as the leading cause of lung cancer development [2, 3]. Non-small cell lung cancer (NSCLC) is the most common type of lung cancer comprising approximately 85 % of lung cancer diagnoses [4]. NSCLC that is discovered early is often treated through resection and adjuvant therapy involving a platinum agent [5]. Advanced disease is treated with palliative platinum based chemotherapy [6]. Only about 10 % of patients with NSCLC will harbor molecular changes rendering their tumor sensitive to an approved targeted agent. While a number of new targeted agents are being investigated, therapies that have novel mechanisms of actions are urgently needed for lung cancer patients [7].

Triptolide is a natural compound isolated from the Thunder God Vine, *Tripterygium wilfordii*, which has been used in traditional Chinese medicine to treat autoimmune disorders and inflammation, including lupus and rheumatoid arthritis [8]. Triptolide also has potent anti-tumor activity in a variety of cancers, including lung cancer [9, 10]. Triptolide perturbs multiple signaling pathways including NFkB, HSP70, and p53 pathways, which decreases cell proliferation and induces apoptosis [11–13]. In lung cancer, triptolide has been shown to sensitize cells to TRAIL-induced apoptosis and enhance p53 activity [14]. We previously showed that triptolide inhibits the Wnt pathway in lung cancer via overexpression of Wnt inhibitory factor 1 (WIF1), which is silenced in most lung cancers by promoter hypermethylation. Though triptolide has anti-tumor effects, its clinical use is limited by toxicity and unfavorable pharmacokinetics [15]. Recently, triptolide derivatives have been developed in order to optimize bioavailability with decreased toxicity.

The triptolide derivative MRx102 (MyeloRx, Vallejo, CA) has been previously shown to have antileukemic activity both in vitro and in vivo by promoting apoptosis of AML cells and can overcome the protection garnered by the microenvironment [16]. Though MRx102 decreases tumorgenicity of blood malignancies, its effect on lung cancer is unknown. To determine the potential of MRx102 as a novel therapeutic for lung cancer, we investigated the effect of MRx102 on the proliferation, survival, and migration of NSCLC cell lines in vitro and the effect on tumor formation and metastasis in vivo. We found that MRx102 significantly decreases Wnt pathway activation, cell proliferation, migration, and invasion in H460 and A549 cells. In addition, both tumor formation and metastasis were inhibited in murine models, including a patient derived xenograft (PDX) NSCLC model.

## Methods

### Reagents and antibodies

Dulbecco’s Modified Eagle Medium (DMEM) was purchased from Life Technologies (Carlsbad, CA). 8.0 micron Transwell dishes with Matrigel coating for invasion assays or without ECM coating for the migration assays were purchased from BD Biosciences (San Jose, CA). MRx102 was a kind gift from MyeloRx LLC (Vallejo, CA).

WIF1 antibody (MABN722) was purchased from EMD/Millipore (Temecula, CA). The GAPDH, phospho β-catenin, and total β-catenin, and p53 antibodies were purchased from Cell Signaling (Danvers, MA). The phosphor Akt and total Akt antibodies were purchased from Santa Cruz Biotechnology (Dallas, TX). HRP-conjugated goat anti-rabbit and goat anti-mouse secondary antibody were purchased from Genetex (Irvine, CA).

### Cell culture and drug treatment methods

H460 and A549 human NSCLC cells were acquired from ATCC and cultured in 5 % CO_2_ at 37 °C in DMEM containing 10 % FBS, 1 % sodium pyruvate, 1 % L-glutamine/gentamycin and 1 % penicillin/streptomycin (complete medium).

MRx102 (MyeloRx, Vallejo, CA) was diluted with DMSO and a 10nM concentration for use in the in vitro assays unless otherwise indicated.

### Annexin V staining for apoptosis

Analysis of apoptosis was conducted using the AnnexinV-FITC Apoptosis Detection Kit from Life Technologies (Carlsbad, CA) according to the manufacturer’s protocol. Briefly, control and triptolide treated H460 and A549 cells were harvested after 48 h and washed with PBS and Binding Buffer. The cells were then labeled with AnnexinV-FITC for 15 min and washed and resuspended in Binding Buffer. Propidium Iodide staining solution was added to the resuspended cells to check for viability. Stained cells were examined using a CyAn flow cytometer (Beckman Coulter, Brea, CA). The FlowJo analysis software was used to analyze the percentage of cells undergoing apoptosis.

### RT-PCR

RNA was isolated using the Purelink RNA Mini kit from Life Technologies (Carlsbad, CA) according to the manufacturer’s protocol. The RNA was then reverse transcribed to cDNA using the High-Capacity cDNA Reverse Transcription kit from Applied Biosystems (Grand Island, NY) according to the manufacturer’s protocol. The RT-PCR was performed using Taqman gene-specific probes (Applied Biosystems) with Taqman Fast Universal Master Mix (Life Technologies) according to the published protocol using the Viia7 RT-PCR machine (Applied Biosystems). The GAPDH RNA expression was used to normalize the WIF1 levels.

### Western blotting

Immunoblotting was performed using nitrocellulose membranes and 4–12 % Bis-Tris Nupage gels from Life Technologies (Carlsbad, CA). The membranes were blocked with 5 % non-fat milk before the addition of the primary antibody.

### Migration and invasion assays

Migration was analyzed using Transwell filters coated with 5 μg/ml fibronectin on the bottom of the filter (haptotaxis). Control or MRx102 treated cells (1×10^5^) were added to the top of the filter and allowed to migrate for 6 h. Cells remaining on top of the filter were removed. The migrated cells were fixed in 4 % paraformaldehyde and the filter was mounted in Prolong Gold with DAPI on a microscope slide. Migration and invasion was analyzed using fluorescence microscopy. The assay was completed three times in triplicate and nine random images were obtained per filter. Invasion assays were performed as previously described and DAPI was used to visualize the cellular nuclei [17].

### Top flash luciferase assay

Transient transfections were performed with polyethylenimine transfection reagent (Sigma, St. Louis, MO) on 1×10^5^ H460 and A549 cells that were plated in a 24-well plate. In the corresponding wells, 0.5 μg of the TOP-FLASH or FOP-FLASH firefly luciferase reporter plasmid and, as an internal control, 0.05 ug of the *Renilla* luciferase reporter pTK (Promega, Madison, WI) was used. After 24 h, DMSO as a control or 10nM MRx102 was added to the corresponding wells. After 48 h of treatment, the cell lysate was collected and the luciferase activity was determined using the Dual Luciferase Assay System (Promega, Madison, WI) and a luminonmeter. The firefly luciferase activity was normalized to the Renilla luciferase activity.

### Bisulfite conversion of genomic DNA and methylation analysis

Genomic DNA from control and MRx102 treated (96 h) A549 and H460 cells was extracted using the QIAamp DNA mini-kit (Qiagen, Valenica, CA) according to the manufacturer’s protocol. 400 ng of the genomic DNA was used for bisulfite conversion. The bisulfite conversion was carried out using the EZ DNA Methylation-Lightning kit (Zymo Research, Irvine, CA) according to the manufacturer’s published protocol. 5 ul of the bisulfite converted DNA was used for PCR analysis with primers specific for the methylated and unmethylated versions of the WIF1 promoter region. The PCR product was then run on a 2 % agarose gel and imaged using UVP Gel Imaging System (Upland, CA).

Primer Sequences:

- **WIF1-methylatedF,**TCGTAGGTTTTTTGGTATTTAGGTC
- **WIF1-methylatedR,**ATACTACTCAAAACCTCCTCGCT
- **WIF1-unmethylatedF,**TGTAGGTTTTTTGGTATTTAGGTTG
- **WIF1-unmethylatedR,**CATACTACTCAAAACCTCCTCACT

### Microscopy

Fluorescence and brightfield imaging were performed using a Zeiss Axio Observer Z1 inverted microscope equipped with Axiocam MRc5 (brightfield) and Hamamatsu Orca CCD (fluorescence) cameras.

### Animal studies

Subcutaneous Xenograft Mouse Model *-* H460 human lung cancer cells (5×10^5^) were injected into the hind flank of 4–8 week old NSG mice. The mice were monitored for tumor growth. Treatment was started when tumors reached 50–100 mm^3^ by measurement with calipers. Mice were split into groups of at least nine mice and treated as indicated with either control (PBS) five times per week, triptolide (0.5 mg/kg) three times per week, MRx102 (1, 2, 3, or 4 mg/kg) five times per week, carboplatin (15 mg/kg) once per week, or a combination of MRx102 (2 mg/kg) and carboplatin (15 mg/kg) once per week, by interperitoneal injection (IP). Tumors were harvested when the tumors in the control group began to reach 1500 mm^3^ (approximately two and half weeks).

Patient-Derived Xenograft Mouse Model *–* Human lung cancer tissue was obtained from research participants at the time of surgical resection of lung cancer. The tissue was collected fresh and was immediately dissected, minced into tissue blocks at about 3 mm in diameter and placed in saline with antibiotics. NSG mice at 6–10 weeks old were anesthetized by isoflurane inhalation. The dorsal area of NSG mice was shaved and prepared with a povidine-iodine/alcohol solution. A small cut was made in the prepared skin and a pocket under skin was created using a pair of forceps. The human cancer tissue blocks were transplanted into this subcutaneous dorsal skin compartment of the NSG mice. The wound was closed by using skin glue. Once the tumors reached a sufficient size, the tissue was passaged into another group of NSG mice. On the third passage, and once tumors reached 100 mm^3^ (as measured by calipers) treatment was started as indicated with either control (PBS) five times per week, MRx102 (3 mg/kg) five times per week, cisplatin (6 mg/kg) once per week, or a combination of MRx102 (3 mg/kg) and cisplatin (6 mg/kg) once per week, by IP injection with at least seven mice per treatment group. Tumors were harvested when control tumors began to approach the 1500 mm^3^ maximum (approximately 3 weeks). Tumors were stained for Wnt3a expression by a participating pathologist (SWT). One high Wnt3a and one low Wnt3a lung adenocarcinoma PDX model was selected for these experiments.

Tail Vein Injection Mouse Model *-* H460 (5×10^4^) and A549 (1×10^5^) cells were injected into the tail vein of 6–8 week old NSG mice. After 2 weeks the mice began to receive control (PBS) or MRx102 (3 mg/kg) by IP injection three times a week for 8 weeks with at least eight mice per group. Mice were then euthanized and the lungs and liver were harvested, fixed in 10 % formalin, and paraffin embedded for pathological examination of H&E slides.

The NSG mice used for these studies were bred at the City of Hope Medical Center animal facility.

### Statistical analysis

All quantified data were plotted and analyzed in GraphPad Prism 6.0 using a Student *t*-test or one-way Anova with Tukey post test. Data are representative of at least 3 independent experiments as replicate means ± SEM. ** or *** are *p* values < 0.01, or 0.001, respectively.

## Results

### MRx102 decreases NSCLC cell proliferation and colony formation and increases apoptosis

**Fig. 1:**
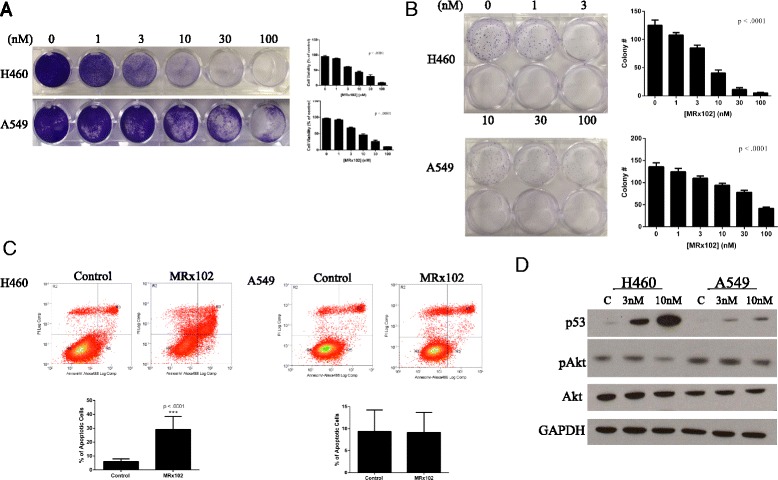
MRx102 decreases lung cancer cell proliferation and increases apoptosis. **a** Cell proliferation assay and corresponding quantification of cell number for H460 (top panel) and A549 (bottom panel) cells treated with MRx102 (0-100nM). **b** Colony formation assay with quantification of colony number for H460 (*top*) and A549 (*bottom*) cells treated with MRx102 (0, 1, 3 nM top row and 10, 30, 100nM bottom row). Annexin V/PI staining of H460 (**c**-*left*) and A549 (**c**-*right*) cells. Quadrants are as follows: Top-left – dead/necrotic cells, Top-right – Late apoptotic cells, Bottom-right – Early apoptotic cells, and Bottom-left – Live cells. **d** Western blot analysis of p53 expression and Akt activation in H460 and A549 cells. *** represents a *p* value of <0.001 as determined by a Student *T*-test

Triptolide has antiproliferative effects in a variety of cancer cell lines [9]. To assess the ability of the triptolide derivative MRx102 to decrease lung cancer cell proliferation, we treated H460 and A549 human NSCLC cells with increasing concentrations of MRx102. After 48 h, MRx102 significantly decreases both H460 and A549 lung cancer cell proliferation in a concentration dependent manner (Fig. 1a). To further study the effectiveness of MRx102 on the proliferation and survival of lung cancer cells, we conducted a colony formation assay with varying concentrations of MRx102. Colony formation of A549 and H460 cells was significantly inhibited by MRx102 treatment with a decrease in both colony number and size as the concentration of MRx102 was increased (Fig. 1b). Next, we wanted to determine if treatment with MRx102 sensitizes cancer cells to apoptosis. After treatment with 10nM MRx102 for 48 h, the percentage of early apoptotic (high Annexin V-488 and low PI staining), late apoptotic (high Annexin V-488 and high PI staining), and necrotic (low Annexin V-488 and high PI staining) cells was determined using FACS analysis. When compared to control cells treated with DMSO, the MRx102 treated H460 cells had significantly higher populations of early apoptotic, late apoptotic, and necrotic cells (Fig. 1c, left). This effect was less notable in the A549 cell line suggesting that MRx102 has less of an effect on apoptotic programming in this cell line or a longer incubation period with the drug is needed to see an effect (Fig. 1c, right). Due to the increase in apoptosis, we looked at p53 and Akt activation, which are known to be involved in cell death and survival. There was a dose dependent increase in p53 expression after MRx102 treatment and slight decrease in phospho Akt levels after treatment at a 10nM concentration of MRx102 (Fig. 1d). This data suggests that MRx102 might regulate cell survival and death by modulation of the p53 and Akt pathways. The decrease in cell proliferation and increase in apoptosis in vitro led us to evaluate the effect of MRx102 on lung cancer cell growth in vivo.

### NSCLC tumor formation and growth is inhibited by MRx102

**Fig. 2:**
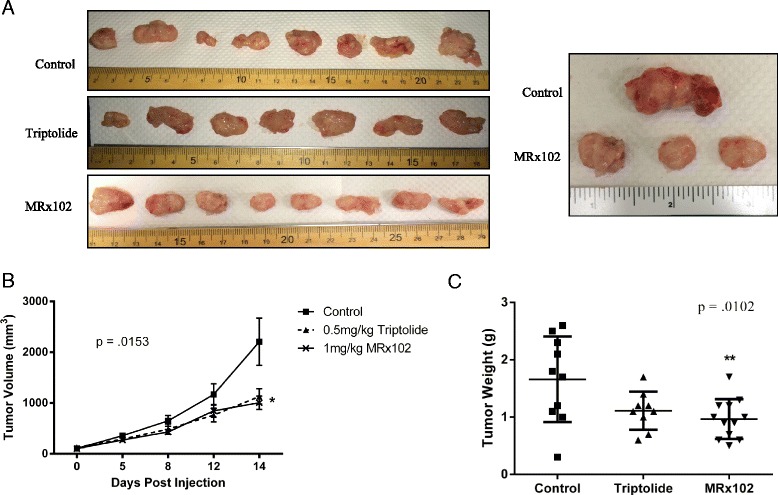
MRx102 inhibits lung cancer tumor formation comparable to triptolide. **a** Mice were subcutaneously injected with H460 cells. Representative tumors from NSG mice that were treated with either PBS (control), 0.5 mg/kg triptolide, or 1 mg/kg MRx102. **b** Quantification of tumor volume from mice in (A). **c** Quantification of tumor weight from mice in (A). No less than 7 mice were used and *p*-values were determined using one-way Anova with a Tukey post test. * and ** represent *p* values of < .05 and <0.01, respectively, and are derived from a comparison between the control and treated groups

To investigate the effect of MRx102 on lung cancer cell growth in vivo, we subcutaneously injected H460 human NSCLC cells in the hind flank of 6–8 week old NSG mice. After treatment with 1 mg/kg MRx102, there was a significant decrease in both tumor volume (*p* = .0153) and weight (*p* = .0102) when compared to vehicle control mice (Fig. 2a, b and c). It has been shown that triptolide treatment can lead to the decrease in tumor formation in mice [18]. The decrease in tumor burden by MRx102 after subcutaneous H460 injection was similar to the effects of triptolide treatment (Fig. 2b). This data suggests that MRx102 has anti-tumor activity in vivo that is comparable to the effects already seen in studies using triptolide.

### MRx102 has additive effects with Carboplatin in the xenograft mouse model

**Fig. 3:**
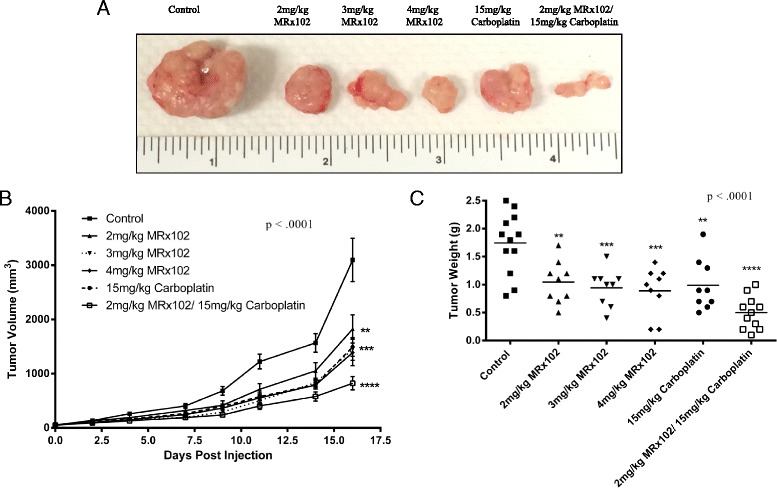
Treatment of xenograft lung cancer cells with MRx102 and carboplatin has an additive effect. **a** Representative tumors from NSG mice injected with H460 cells and treated as indicated. **b** Tumor volume measurements from NSG mice (A) treated with varying concentrations of MRx102, carboplatin, or a combination of MRx102 and carboplatin. **c** Quantitative measurement of tumor weight from NSG mice (A) treated as indicated. No less than 9 mice were used and *p*-values were determined using one-way Anova with a Tukey post test. **, ***, and **** represent *p* values of <0.01, <.001, and < .0001, respectively, and are derived from a comparison between the control and treated groups

Platinum agents, including carboplatin and cisplatin, have been used as standard components of NSCLC chemotherapeutic regimens [5]. Based on the ability of MRx102 to decrease NSCLC tumor formation in mice, we treated mice harboring subcutaneous H460 tumors with increasing concentrations of MRx102 (2, 3, and 4 mg/kg), 15 mg/kg carboplatin, or a combination of 2 mg/kg MRx102 and 15 mg/kg carboplatin to compare the efficacy of MRx102 with the standard NSCLC platinum therapy (Fig. 3a). Treating with 2, 3 or 4 mg/kg MRx102 had similar effects compared to treating with 15 mg/kg carboplatin decreasing tumor size by more than half (*p* < .0001) (Fig. 3b) and reducing tumor weight (*p* < .0001) (Fig. 3c). When combined, 2 mg/kg MRx102 and 15 mg/kg carboplatin appeared to have an additive effect on decreasing tumor formation in mice (Fig. 3b and c).

### MRx102 treatment inhibits lung cancer growth in a low WIF1 expressing PDX model of NSCLC

**Fig. 4:**
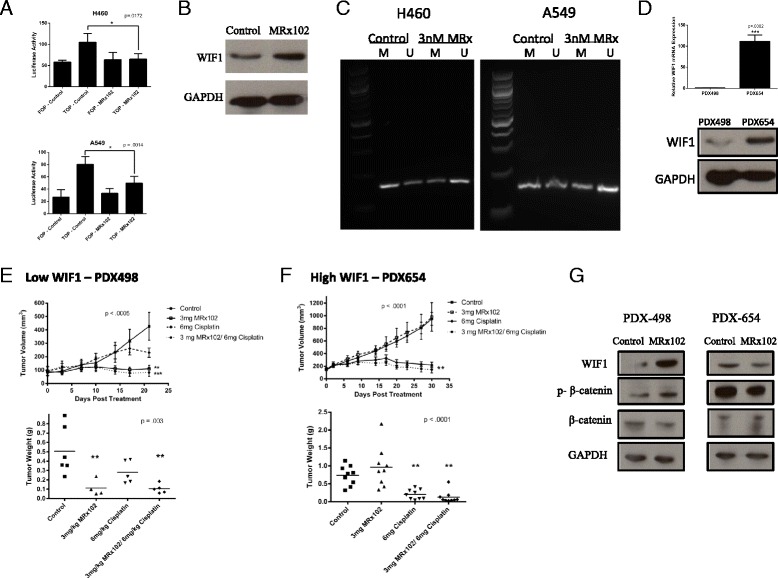
Patient tumors expressing high Wnt3a levels are sensitive to MRx102 treatment. **a** TOP-Flash Wnt activity assay in MRx102 treated H460 (*left*) and A549 (*right*) cells. **b** Western blot analysis of WIF1 expression in H460 mouse xenograft tumors. **c** WIF1 promoter methylation status in H460 and A549 cells treated with MRx102. **d** RT-PCR (*top*) and western blot (*bottom*) analysis of WIF1 expression in the patient-derived xenograft tissue. **e** Patient tumor expressing low WIF1 shows decreased tumor volume (*top*) and weight (*bottom*) when treated with 3 mg/kg MRx102 compared to 6 mg/kg cisplatin treatment alone. **f** High WIF1 expressing patient tumor does not respond to MRx102 treatment. Tumor volume (*top*), tumor weight (*bottom*). n = 7. **g** Western blot analysis of WIF1, phospho-β-catenin, and total β-catenin in the patient-derived xenograft tissue treated with 3 mg/kg MRx102 compared with control tumors. *p* values were determined using a Student t-test or one-way Anova with a Tukey post test. *, **, and *** represent *p* values of < .05, <0.01, and < .001, respectively, and are derived from a comparison between the control and treated groups

Wnt pathway alterations significantly contribute to lung cancer progression and overexpression of Wnt pathway molecules has been associated with poor prognosis in NSCLC [19, 20]. We previously found that triptolide inhibits Wnt pathway activity through increased WIF1 expression. To test the affect of MRx102 on Wnt pathway activity, we conducted a TOP-Flash luciferase assay. In both H460 and A549 cells, luciferase activity decreased after treatment with MRx102 suggesting that treatment with this drug decreases Wnt pathway activation (Fig. 4a). Because we found that treatment with MRx102 decreases Wnt pathway activation in NSCLC cell lines, we next analyzed the tumors of our previous mouse xenograft experiment for the expression of WIF1. We found that the tumor tissue from mice that had been treated with MRx102 had significantly increased WIF1 expression (Fig. 4b). We further wanted to elucidate a possible mechanism for the increase in WIF1 expression. As we previously stated, we found that triptolide decreases WIF1 promoter methylation, so we conducted PCR using WIF1 methylated and unmethylated specific primers with bisulfite converted genomic DNA. In both A549 and H460 cells, treatment with MRx102 decreased methylation at the WIF1 promoter and increased the unmethylated population (Fig. 4c). This data suggests that MRx102 increases WIF1 expression by epigenetic modulation.

To investigate the efficacy of MRx102 on patient tumor tissue, we subcutaneously implanted NSCLC tissue from patients with either low or high WIF1 expressing tumors by RT-PCR and western blot analysis into NSG mice (Fig. 4d). We found that the patient tumors that expressed low WIF1 levels were sensitive to 3 mg/kg MRx102 (*p* < .0005) while there was little effect of MRx102 treatment in a high WIF1 expressing PDX (Fig. 4e and f). In the low WIF1 expressing tumors, MRx102 both alone and in combination with cisplatin treatment had a greater effect than cisplatin treatment alone (Fig. 4e). Finally, after treatment with MRx102, there was an increase in WIF1 and phospho-β-catenin protein expression in the low WIF1 expressing PDX suggesting that there is a decrease in Wnt pathway activity after treatment (Fig. 4g). These data suggests that MRx102 could have a positive impact on patients whose tumors have low WIF1 expression levels and that Wnt pathway activity could be used as a biomarker for the use of MRx102 as a targeted therapy. These data also indicates that low WIF1 expression may be an important marker for sensitivity to MRx102 effects in lung cancer patients.

### Migration and Invasion of NSCLC cells is inhibited by MRx102 treatment

**Fig. 5:**
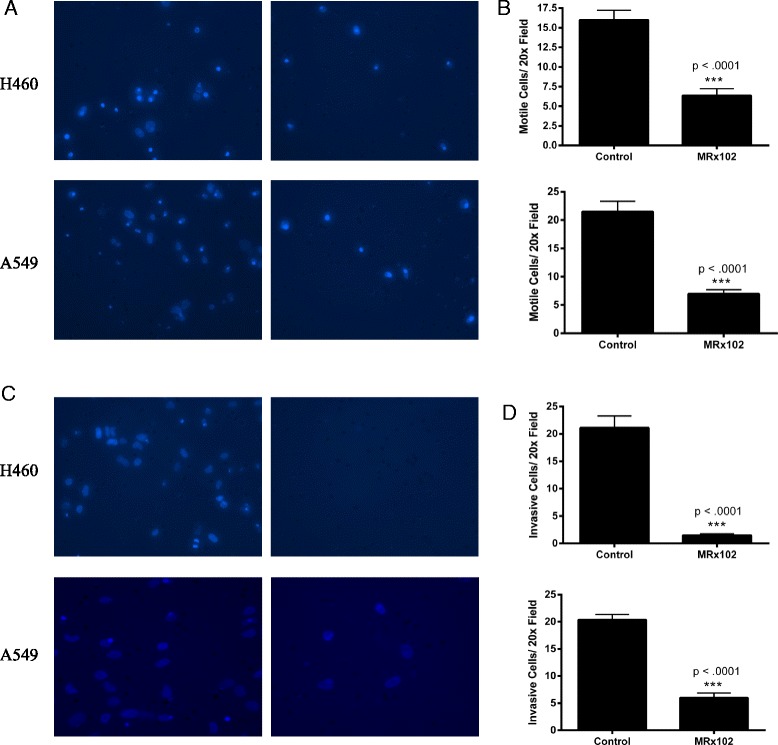
MRx102 decreases lung cancer cell migration and invasion. **a** Representative images of migrated H460 (*top*) and A549 (*bottom*) cells treated with either DMSO control (*left*) or 10nM MRx102 (*right*) stained with Prolong Gold with DAPI. **b** Quantitative measurement of migrated cells in (A). **c** Representative images of invasive H460 (*top*) and A549 (*bottom*) cells treated with either DMSO control (*left*) or 10nM MRx102 (*right*) stained with Prolong Gold with DAPI. **d** Quantitative measurement of invasive cells from (C). *n* = 27 20x fields per group. *** represents a *p* value of <0.001 as determined by a Student t-test

To study if MRx102 has an effect on lung cancer cell migration, we plated H460 and A549 cells that had been treated with 10nM MRx102 for 48 h on fibronectin coated Transwell filters and allowed the cells to migrate for 6 h. Cells treated with MRx102 migrated significantly less than the DMSO control cells (Fig. 5a and b). The adhesion of both the treated and control cells to fibronectin was similar indicating that MRx102 does not alter the ability of cells to adhere to the extracellular matrix (data not shown).

Cancer progression is dependent upon the ability of cells to degrade and invade the extracellular matrix components surrounding the tumor [21]. To investigate if MRx102 can alter lung cancer cell invasion, we used Matrigel coated Transwell chambers and allowed the NSCLC cells to invade for 24 h. We found that MRx102 significantly decreases lung cancer cell invasion through Matrigel compared to the control cells (Fig. 5c and d). This data suggests that MRx102 could potentially inhibit cancer cell migration and invasion in vivo.

### MRx102 decreases NSCLC metastasis in mice

**Fig. 6:**
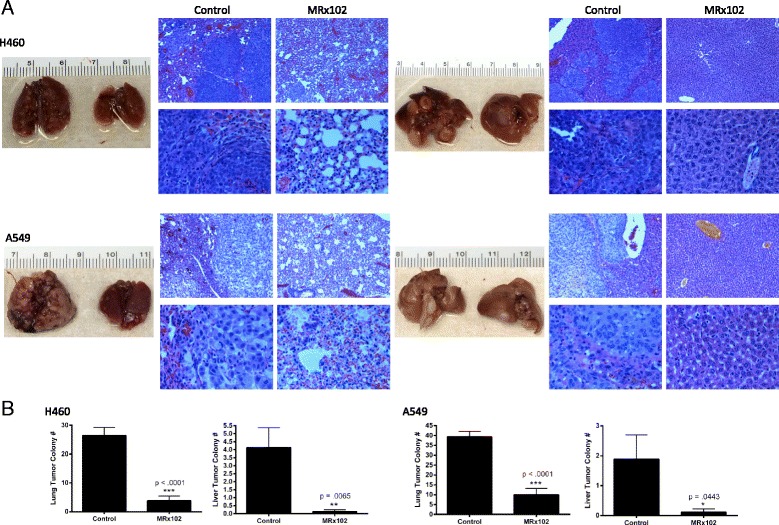
Metastatic lesion formation is inhibited by MRx102 treatment. **a** Representative whole organ imaging and H&E tissue staining of NSG mice injected with H460 (top grouping) and A549 (bottom grouping) cells. Lung (*left*) and liver (*right*) images with corresponding pathological analyses. In each H&E stain grouping top-left – control (PBS) treatment at 5x magnification, bottom-left - control (PBS) treatment at 20x magnification, top-right – MRx102 (3 mg/kg) treatment at 5x magnification, and bottom-right – MRx102 (3 mg/kg) treatment at 20x magnification. **b** Quantification of H460 (left grouping) and A549 (right grouping) metastatic colony formation in lung (*left*) and liver (*right*) from tail-vein injected NSG mice. *n* = 8 mice, *, **, and *** represents *p* values of < .05, <.01, and <0.001 as determined by a Student t-test

Because both lung cancer cell migration and invasion are significantly altered by MRx102 treatment in vitro, we next wanted to examine the effect of MRx102 treatment on metastasis in vivo. NSG mice injected with H460 and A549 cells through the tail vein were used as a model for lung cancer cell metastasis. Mice treated with 3 mg/kg MRx102 had significantly less metastatic lesion formation in the lungs (*p* < .0001) and liver (*p* = .0065 and *p* = .0443) than the vehicle control mice (Fig. 6a and b). This data indicates that MRx102 could be a potential lung cancer therapeutic for targeting lung cancer progression.

## Discussion

Triptolide is a natural product that has been shown to be an effective anti-inflammatory and anti-tumor compound [8]. Though triptolide has potent effects in pre-clinical studies, the development of triptolide into a useful cancer drug has been limited by its unfavorable pharmacokinetic and toxicity profile [15]. The toxicity of triptolide has led to the development of derivatives that are being tested for having a similar effect as triptolide without the unwanted toxicity. One of these triptolide derivatives is MRx102. MRx102 is converted to triptolide during in vivo treatment and was found to have a similar mechanism of action compared to triptolide [16]. Here we have shown that MRx102 significantly decreases NSCLC cell proliferation, migration, and invasion. MRx102 also leads to increased apoptosis in H460 cells through upregulation of p53 and decreased Akt activity. In addition to its anti-proliferative effects in vitro, MRx102 is also effective in reducing tumor formation and metastasis in NSG mice injected with NSCLC cells.

Canonical Wnt pathway signaling is crucial for proper embryonic development by controlling cell migration, proliferation, and differentiation [22]. Wnt pathway components also facilitate the maintenance of the adult stem cell population [23]. Aberrant activation of these components and maintenance of the stem cell populations is thought to contribute to the carcinogenesis of many malignancies including NSCLC [24, 25]. Promoter hypermethylation and downregulation of WIF1, a secreted Wnt inhibitor that modulates Wnt activity by directly binding to Wnt ligands, is thought to be critical for lung cancer progression and is a prognostic biomarker for patient survival [26]. We found that MRx102 decreases Wnt pathway activation and increases WIF1 expression. Notably, we determined that the increase in WIF1 expression was due to changes in epigenetic modification at the WIF1 promoter region. Importantly, we discovered that MRx102 has the greatest efficacy in lung cancer cell lines with low WIF1 expression. Also patient tissue that has low expression of WIF1 was more sensitive to MRx102 treatment than tumors with high WIF1 expression. The ability of MRx102 to target the Wnt pathway and the sensitivity of WIF1 downregulated tumors to MRx102 treatment could lead to MRx102 as a viable therapeutic for patients who have low expression of this inhibitory factor.

We did not observe any adverse effects, including no change in weight or activity level, with 2–4 mg/kg MRx102 treatment administered by IP injection 5 days a week. The time lengths of treatment varied from 3 to 8 weeks, so it is possible that there may be treatment-related adverse effects during prolonged treatment. Taken together, MRx102 appears to be well tolerated at higher doses compared to triptolide.

## Conclusion

In summary, we found that MRx102 treatment decreases lung cancer cell proliferation, tumor formation, and metastasis and can work synergistically with platinum based therapy. We have also shown that cell lines and patient tissue with low WIF1 expression are more sensitive to MRx102 treatment than high expressing cells and tissue. These studies establish that MRx102 is a potent anti-tumor agent in lung cancers and is a prospective novel lung cancer therapeutic.

## Abbreviations

NSCLC, non-small cell lung cancer; PDX, patient-derived xenograft; WIF1, Wnt inhibitory factor 1
